# Serving as a bedside surgeon before performing robotic radical prostatectomy improves surgical outcomes

**DOI:** 10.1590/S1677-5538.IBJU.2019.0330

**Published:** 2019-12-17

**Authors:** Haci Ibrahim Cimen, Yavuz Tarik Atik, Deniz Gul, Burak Uysal, Mevlana Derya Balbay

**Affiliations:** 1 Department of Urology, Training and Research Hospital, Sakarya University, Turkey; 2 Department of Urology, American Hospital, Koc University, Turkey

**Keywords:** Prostatic Neoplasms, Prostatectomy, Robotics

## Abstract

**Introduction:**

To evaluate the influence of previous experience as bedside assistants on patient selection, perioperative and pathological results in robot assisted laparoscopic radical prostatectomy.

**Materials and Methods:**

The first 50 cases of two robotic surgeons were reviewed retrospectively. Group 1 consisted of the first 50 cases of the surgeon with previous experience as a robotic bedside assistant between September 2016-July 2018, while Group 2 included the first 50 cases of the surgeon with no bedside assistant experience between February 2009-December 2009. Groups were examined in terms of demographics, prostate volume, presence of median lobe, prostate specific antigen (PSA), preoperative Gleason score, positive core number, clinical stage, console surgery time, estimated blood loss, postoperative Gleason score, pathological stage, positive surgical margin rate, postoperative complications, length of hospital stay and biochemical recurrence rate.

**Results:**

Previous abdominal surgery and the presence of median lobe hypertrophy rates were higher in Group 1 than in Group 2 (20% vs. 4%, p=0.014; 24% vs. 6%, p=0.012; respectively). In addition, patients in Group 1 were in a higher clinical stage than those in Group 2 (cT2: 70% vs. 28%, p=0.001). Median console surgery time and median length of hospital stay was significantly shorter in Group 1 than in Group 2 (170 min vs. 240 min, p=0.001; 3 vs. 4, p=0.022; respectively). Clavien grade 3 complication rate was higher in Group 2 but was statistically insignificant.

**Conclusion:**

Our findings might reflect that previous bedside assistant experience led to an increase in self-confidence and the ability to manage troubleshooting and made it more likely for surgeons to start with more difficult cases with more challenging patients. It is recommended that novice surgeons serve as bedside assistants before moving on to consoles.

## INTRODUCTION

Robotic surgery continues to advance and promises to play an increasingly large role in the field of urology. The advantages of this technology, such as low perioperative blood loss, low postoperative pain, short hospital stays, and faster patient recovery, has made it more common in prostate cancer treatment (1). However, its use has also lead to questions about how best to improve the skills of robotic surgeons and which training methods are most suitable for skill acquisition.

In the last two decades, urologic oncology surgery training in minimally invasive and robotic techniques has become more important. Therefore, various training models, such as wet/dry laboratories (2), animal models or human cadavers (3), virtual reality simulators (4, 5) and mentorship programmes (6) have been utilized.

During open surgery, the mentor and surgeon actively collaborate in the operative field. However, in robot assisted laparoscopic radical prostatectomy (RALRP), only one person can seat at the console, and if it is not a dual console system, the mentor guides the console surgeon verbally to improve his or her learning curve. If the surgeons have prior experience in robotic surgery and become familiar with the difficulties and tricks of the surgery earlier, the learning curve for robotic surgery might be positively affected and improve (7). However, there exists a lack of adequate data regarding the impact of console surgeon's previous bed-side assistance experience on the learning curve and their choice for more challenging patients for their initial surgeries with RALRP. The aim of the current study is to evaluate whether the previous experience of console surgeons as bed-side assistants affects their patient selection and perioperative and oncological results in RALRP.

## MATERIALS AND METHODS

After local ethics committee approval, the first 50 cases of two robotic surgeons were reviewed retrospectively. Group 1 included the first fifty cases of a surgeon who had robotic bedside assistance experience (at least 150 cases) between September 2016-July 2018, while Group 2 included the first fifty cases of a surgeon who had open radical prostatectomy experience (at least 300 cases) but no bedside assistance experience between February 2009-December 2009. The cases of both surgeons were completed with the same transperitoneal approach with 5 ports (1 camera, 3 robotics and 1 assistant).

Demographic data were included: age, comorbidities, history of abdominal and prostate surgery, preoperative prostate specific antigen (PSA) levels, Gleason scores and positive cores in transrectal ultrasound prostate biopsy, prostate volume, presence of median lobe and clinical stage. Median lobe was defined as any prostatic tissue protruding into the bladder neck anteriorly and searching for the ureteric orifices before completing the posterior neck incision above them. In addition, estimated blood loss, console surgery time, length of hospital stay, oncological data including pathological stage, surgical margin positivity and biochemical recurrence in postoperative first year were also reviewed. Biochemical recurrence was defined as a postoperative PSA value of >0.2ng/mL in the follow-up period which was measured in postoperative 1^st^ month and 3 month intervals in the following period.

The comorbidities of the patients were calculated according to the modified Charlson comorbidity index (MCCI). The console surgery time was measured from the time the surgeon started using the console hand pieces to the time of removal of the instruments from the patient.

Statistical analyses were performed using the SPSS 21.0 (IBM, NY, USA) statistical program. The Kolmogorov-Smirnov test was used to evaluate the appropriateness of data to normal distribution. For variables with non-normal distribution, the medians (min-max) were calculated and displayed. Categorical variables were displayed as number of cases (n) and percentage (%). The variables were grouped when necessary in order to interpret the statistical analysis. We compared continuous data in each group using independent samples t test or Mann-Whitney U test and categorical data using chi-square test or Fisher's exact tests as appropriate. The level of statistical significance was set at P <0.05.

## RESULTS

Both groups consisted of 50 patients. No statistically significant differences were found between the groups regarding age, MCCI, previous prostate surgery rates, prostate volume, PSA levels, preoperative Gleason score and positive core number (Table-1). Previous abdominal surgery and the presence of median lobe hypertrophy rates were higher in Group 1 (20% vs. 4%, p=0.014; 24% vs. 6%, p=0.012; respectively). In addition, patients in Group 1 were in a higher clinical stage (T2: 70% vs. 28%, p=0.001). When we evaluated the perioperative and postoperative results, we found no difference in terms of estimated blood loss, postoperative blood transfusion and complication rates, postoperative Gleason score, surgical margin positivity and PSA recurrence rates (Table-2). Median console surgery time was shorter in Group 1 than in Group 2, and the difference was statistically significant (170 min vs. 240 min, p=0.000, respectively). Furthermore, median length of hospital stay was shorter in Group 1 than in Group 2 (3 vs. 4 days, p=0.022, respectively). However, the pathological result was found to be higher in Group 2 than in Group 1 (pT3: 54% vs. 32%, p=0.026). In Group 1, Clavien grade 3 complication occurred in two patients who were admitted to the intensive care unit in the postoperative period (one for respiratory arrest and the other for acute respiratory distress syndrome). Both of the patients were discharged without any sequela. In Group 2, four patients experienced Clavien grade 3 complications. One patient underwent cystoscopy under general anaesthesia due to spontaneous urethral catheter dislocation. Rectal and bladder injuries occurred in two patients and were repaired perioperatively with running closure of the mucosa and serosa with a 3-0 absorbable suture separately without any permanent sequela. One patient underwent nephrectomy for hydronephrosis as a result of complication due to ureteric ligation at the time of surgery.

**Table 1:** Comparison of preoperative data.

		Group 1 (*n*=50)	Group 2 (*n*=50)	*P*
Age (years) [median (min-max)]		65.5 (51-74)	64 (45-76)	0.407*
MCCI [median (min-max)]		4 (3-6)	4 (2-6)	0.375*
Previous abdominal surgery, *n(%)*		10 (20)	2 (4)	0.014**
Previous prostate surgery, *n(%)*		2 (4)	–	0.495***
Prostate volume (cc) [median (min-max)]		55 (24-120)	50 (18-100)	0.158*
Presence of median lob hypertrophy, *n(%)*		12 (24)	3 (6)	0.012**
PSA (ng/dL) [median (min-max)]		7.6 (6. 8-37.79)	6.43 (1.41-27)	0.461*
**Preoperative Gleason score, *n****(%)*	6	33 (66)	35 (70)	0.436***
	7	15 (30)	10 (20)	
	8-10	2 (4)	5 (10)	
Positive core (n) [median (min-max)]		4 (0-12)	3 (1-12)	0.437*
**Clinical stage, *n***(%)	T1b	1 (2)	0 (0)	0.001**
	T1c	14 (28)	36 (72)	
	T2a	19 (38)	9 (18)	
	T2b	8 (16)	0 (0)	
	T2c	8 (16)	5 (10)	

MCCI = Modified Charlson comorbidity index; PSA = Prostate specific antigen

* = Mann-Whitney U

** = Chi-Square Test

*** = Fisher's Exact Test

**Table 2:** Comparison of peroperative and postoperative data.

		Group 1 (*n*=50)	Group 2 (*n*=50)	P
Console surgery time (min) [median (min-max)]		170 (145-240)	220 (90-380)	0.001*
Estimated blood loss (cc) [median (min-max)]		135 (90-250)	200 (40-1000)	0.485*
**Postoperative Gleason score, *n*(%)**	6	24 (48)	30 (60)	0.678***
	7	23 (46)	12 (24)	
	8-10	2 (4)	4 (8)	
	pT0	1 (2)	4 (8)	
**Pathological stage, *n***(%)	T2a	4 (8)	7 (14)	0.026**
	T2b	2 (4)	1 (2)	
	T2c	27 (54)	11 (22)	
	T3a	11 (22)	21 (42)	
	T3b	5 (10)	6 (12)	
	pT0	1 (2)	4 (8)	
Positive surgical margin, *n(%)*		11 (22)	9 (18)	0.617**
Postoperative blood transfusion, *n(%)*		3 (6)	5 (10)	0.715***
Clavien ≥3 Complications, *n(%)*		2 (4)	4 (8)	0.678***
Length of hospital stay (day) [median (min-max)]		3 (2-10)	4 (3-11)	0.022*
Biochemical recurrence, *n(%)*		5 (10)	10 (20)	0.161**

* = Mann-Whitney U

** = Chi-Square Test

*** = Fisher's Exact Test

## DISCUSSION

The proper utilization of RALRP first requires proper training methods in robotic surgery. The most important aims of robotic surgery training include optimizing surgical outcomes and patient safety and minimising surgical complications in the period of learning. The ideal performance plateau, approximately 200-300 cases, is necessary for superior surgical outcomes, and suboptimal conditions may lead to medico-legal issues (8). For these reasons, training before sitting at the console and introductory courses are becoming more important.

Several virtual reality simulators have been used for various surgical skills and have been shown to positively affect different stages of surgery (9). Dry-lab training is performed with simulator tools that use non-human and non-animal models which have tissue-like synthetic material (10). Benson et al. evaluated the effect of a dry-lab training programme with 43 novice students and reported that ring transfer, suture placement and knot tying manoeuvres significantly improved with use of the procedure (11). Wet-lab exercises refer to the use of animal and cadaveric models, Sierra et al. showed that for training using wet-lab exercises, 69.2% of trainees displayed competence at performing robotic surgery and undertaking their first procedure at their institution (12).

On the other hand, Moglia et al. reported an absence of consensus on the effectiveness of these simulations in skill acquisitions which are transferable when operating on real patients (13). Another limiting factor for using these training models is their high cost, which many institutions find prohibitive (8).

However, there is a lack of adequate data regarding the impact of console surgeon's previous bed-side assistance experience on their learning curves. In our study, we found no difference in terms of estimated blood loss, postoperative blood transfusion and complication rates, postoperative Gleason score, surgical margin positivity and PSA recurrence rates between the two groups. On the other hand, previous abdominal surgery and the presence of median lobe hypertrophy rates were higher in Group 1. Patient selection requires greater attention for the initial cases of RALRP. Skarecky et al. suggested that early cases should have no previous history of abdominal surgery and that surgeons should avoid the challenges of large median lobes (14). In other studies, the presence of a median lobe was a predictor of prolonged bladder neck division and correlated with prolonged operative time (15, 16). Cases of previous abdominal surgery has been associated with worse peri-operative outcomes, and surgeons with more experience are more likely to treat these patients (17). Our results can be explained by the fact that identifying the junction between the bladder neck and the prostate and dissection of adhesions was easier for the Group 1 surgeon because of this surgeon's observance of various tricks and manipulations during his or her bedside assistance, before commencing as a console surgeon.

Robot assisted laparoscopic radical prostatectomy following transurethral resection of prostate (TURP) has been reported as more challenging for robotic surgeons because the extravasation of irrigation fluid leads to fibrosis and worse surgical planes (18, 19). Hung et al. uncovered an increased need for bladder neck reconstruction, longer vesicoureteral anastomosis times, higher major complication rates such as rectal injury, and inferiority about neurovascular bundle preservation, in patients who had previously undergone TURP (20). In our study, two patients in Group 1 had a previous TURP history, but no patients in Group 2 had such a history, the difference was not statistically significant.

Operation time is another important factor in robotic surgery, as we reported in our previous study, it decreases with more experience for both the console surgeon and the bedside assistant (21). Guzzo et al. reported that as the surgeons become more experienced at the bedside, they become more proficient in trocar port placement, docking and undocking, instrumentation and troubleshooting with greater accuracy (22). They also envision themselves performing robotic surgery with greater confidence. They suggested that as the surgeon's technique improved with bedside training, the operation time decreased as the number of performed cases increased. Our results revealed that the console surgery time was significantly lower in Group 1. This result can be explained by the fact that the Group 1 surgeon gained more experience on collisions and trouble-shooting management from observations during his time as a bedside assistant. This observation would have provided crucial details about the specific steps of the surgery.

In certain countries, there is a lack of adequate training in the robotic surgery field and there may be no specialized training center. Therefore, only a few surgeons have the opportunity to train adequately before facing a real case. Most of them never had the opportunity to perform robotic surgeries in swine or cadavers before the first case. This fact may contribute to the divergent outcomes from a considerable percentage of the new robotic surgeons when compared with referral centers worldwide.

Limitations of the present study include its retrospective nature and the relatively small number of patients. Furthermore, clinical examinations of the patients and surgical specimens were not carried out by the same clinicians or pathologists. This may have impacted the difference between groups in terms of clinical and pathological stage. Additionally, the functional outcomes between 2 groups were not compared because of both lack of data and the difference in follow-up period.

## CONCLUSIONS

Our findings might reflect that previous bedside assistance experience led to an increase in self-confidence and the ability to manage troubleshooting for console surgeons. It also increased the likelihood of these surgeons taking on more difficult or challenging patient cases at robotic consoles. We recommend that novice surgeons should serve as bedside assistants before moving on to the console, bedside assistance experience, it is expected, increases their experience and acquaints them with surgical procedure, which, in turn, optimizes surgical outcomes and patient safety and minimises surgical complications during the learning curve.

### Compliance with Ethical Standards

The study had been reviewed and approved by Ethical Board of Sakarya University School od Medicine with number of 71522473/050.01.04/196 and with date of 07/27/2018.
